# Is Organizational Communication Climate a Precondition for Patient-Centered Care? Insights from a Key Informant Survey of Various Health and Social Care Organizations

**DOI:** 10.3390/ijerph17218074

**Published:** 2020-11-02

**Authors:** Kira Isabel Hower, Vera Vennedey, Hendrik Ansgar Hillen, Stephanie Stock, Ludwig Kuntz, Holger Pfaff, Timo-Kolja Pförtner, Isabelle Scholl, Lena Ansmann

**Affiliations:** 1 Institute of Medical Sociology, Health Services Research, and Rehabilitation Science (IMVR), Faculty of Human Sciences and Faculty of Medicine, University of Cologne, 50933 Cologne, Germany; holger.pfaff@uk-koeln.de (H.P.); timo-kolja.pfoertner@uk-koeln.de (T.-K.P.); 2 Institute for Health Economics and Clinical Epidemiology, University Hospital Cologne (AöR), 50935 Cologne, Germany; vera.vennedey@uk-koeln.de (V.V.); stephanie.stock@uk-koeln.de (S.S.); 3 Department of Business Administration and Health Care Management, University of Cologne, 50931 Cologne, Germany; hhillen@gmx.net (H.A.H.); kuntz@wiso.uni-koeln.de (L.K.); 4 Department of Medical Psychology, University Medical Center Hamburg-Eppendorf, 20246 Hamburg, Germany; i.scholl@uke.de; 5 Department of Health Services Research, Faculty of Medicine and Health Sciences, Carl von Ossietzky University Oldenburg, 26129 Oldenburg, Germany; lena.ansmann@uni-oldenburg.de

**Keywords:** patient-centered care, implementation, communication, health and social care organizations, decision-maker

## Abstract

Health and social care organizations are under pressure of organizing care around patients’ needs and preferences while complying with regulatory frameworks and constraint resources. To implement patient-centered care in health and social care organizations successfully, particular organizational preconditions need to be considered. Findings on the implementation of patient-centered care and its preconditions are rare and insufficiently account for the organizational context to explain differences. This study examines the implementation status of patient-centered care in diverse health and social care organizations and analyzes the communication climate as a precondition of successful implementation. In a cross-sectional postal key informant survey, decision makers in the highest leading positions from six different types of health and social care organizations in Cologne, Germany, were surveyed using a paper–pencil questionnaire. Patient-centered care implementation was operationalized by three categories (principles, activities, and enablers) including 15 dimensions. Organizational communication climate was operationalized by aspects of open and constructive communication, cooperation, and inclusion. Out of 1790 contacted organizations, 237 participated. In the analyses, 215 complete datasets were included. Descriptive analyses, Kruskal–Wallis test, post hoc pair-wise test, and linear regression modeling were performed. Results show that the implementation status of patient-centered care was perceived as high but differed between the various types of organizations and in terms of patient-centered care categories. Organizational communication climate was significantly associated with the implementation of patient-centered care. Especially in organizations with a higher number of employees, strategies to create a positive communication climate are needed to create a precondition for patient-centered care.

## 1. Introduction

Patient-centered care (PCC) has become a guiding principle in health and social care and is defined as ‘providing care that is respectful of, and responsive to, individual patient preferences, needs and values, and ensuring that patient values guide all clinical decisions’ [1].

These developments put health and social care organizations (HSCOs) under pressure to develop strategies to implement PCC while complying with regulatory frameworks and constraint resources. The extent to which PCC is considered and implemented successfully depends on various organizational preconditions [2,3]. Studies show that the implementation varies between HSCOs [4]. This raises the question about organizational preconditions of HSCOs that determine PCC implementation [5,6].

Research has identified several preconditions for successful PCC implementation relating to HCSO’s processes, structures, strategies, culture, and characteristics of providers [7]. However, the results are usually limited to specific aspects of PCC or specific types of HSCOs, especially hospitals or hospital departments [7,8]. The methodological approaches also vary, with qualitative approaches dominating [4,7,9,10].

Despite this heterogeneity, certain overarching organizational requirements have been identified. These have also been compiled in our own work, where organizational preconditions for PCC implementation were examined in a comprehensive, explorative, qualitative approach across different HSCO contexts [9]. As in other studies [5,10], organizational communication climate (formal and informal, between internal or external stakeholders) was found to be fundamental for PCC implementation and patient outcomes. Organizational communication climate is an atmosphere and an expression of social interaction, harmony, and inclusion, and not only cooperation or an exchange of information between organizational members [11]. It is based on high social capital and enables social cooperation, psychological safety, and trustful debates over problems at work [12,13]. A positive organizational communication climate is, for example, characterized by an understanding of roles and responsibilities, agreement on the approach to care, absence of power dynamics, proper communication patterns, and constant exchange of information [14]. A lack of these characteristics might contribute to a work environment, in which errors, risks, oversights, and deviations are not openly communicated. As a consequence, organizational communication climate was found to negatively affect patient outcomes [14,15,16] such as patient satisfaction [17,18,19], patient safety [12,16,19], complications, or death [20]. Another consequence of a positive open organizational communication climate might be healthy, supportive relationships, and a good well-being of employees, which in turn are strongly associated with the implementation of PCC [9,19].

Consequently, organizational communication climate needs to be considered as a crucial precondition for PCC implementation in research and practice [9,11]. However, studies about the association of organizational communication climate and PCC are lacking [11,21]. Furthermore, there are differences in terms of communication climate and PCC in dependence of HSCO settings, which restricts the generalizability of the relevance of communication climates across various HSCOs for PCC. Previous studies have rarely taken into account the differences between different types of HSCOs in investigating PCC implementation and associations with organizational communication climate [11,21]. Our study aims to address these gaps and examines the self-reported implementation status of PCC within diverse HSCOs, analyzes the organizational communication climate as precondition for PCC implementation, and tests whether the association between communication climate and PCC implementation differs by HSCO type.

## 2. Materials and Methods

### 2.1. Study Design

The study presented in this paper is part of the larger research project OrgValue (Characteristics of Value-Based Health and Social Care from Organizations’ Perspectives). OrgValue is embedded within the Cologne Care Research and Development Network (CoRe-Net) that connects practice and scientific research and aims to improve patient-centeredness and value-based care for chronically ill patients in the metropolitan region of Cologne, Germany [22]. OrgValue analyses the implementation of PCC while considering the HCSOs’ preconditions and strategies towards the implementation with a mixed methods approach [23]. This paper presents results of the quantitative survey of several types of HSCOs in the region of Cologne, which are involved in the care of chronically ill patient groups studied within CoRe-Net. The Ethics Committee of the Medical Faculty of the University of Cologne approved the study (reference number: 17–210).

### 2.2. Data Collection

Study design and participants: The implementation status and organizational preconditions for PCC were surveyed from the perspectives of key informants from hospitals, rehabilitation facilities, outpatient and inpatient nursing facilities, psychotherapy practices, general practitioners (GPs), and cardiological/internal specialist practices in the city of Cologne, Germany. A key informant survey was chosen because it enables a substantially larger number of organizations to be surveyed at lower cost [24]. Inclusion criteria to participate as a key informant were that the persons were in the highest leading clinical or managerial position within the relevant types of HSCO with decision-making authority out of the statutory health and social care sector. Insufficient German language skills to take part in the survey were an exclusion criterion.

Recruitment and data collection: Contact information (registered type and designation of HSCO, address and name of the leading decision makers) was gathered from the Association of Statutory Health Insurance Physicians and own web-based research. The total population of the study were 1790 organizations. The HSCOs were contacted by post with study information accompanied by an informed consent form, the paper–pencil questionnaire, and prepaid return envelopes in accordance with the “Total Design Method” [25] including two personal reminders and considering respective design and layout aspects. Non-responders were followed up with a reminder postcard three weeks after the initial send out, and with replacement questionnaires after another three weeks. Moreover, several strategies shown to increase survey response rates were applied (e.g., personalized letters, prepaid envelopes, highlighting the academic origin, participation in events of organizational learning, and survey feedback via anonymous benchmarking reports). As an incentive, a donation of 1€ per completed questionnaire to a charity organization for disadvantaged children in the city of Cologne was advertised.

### 2.3. Measures

The questionnaire development used in this study draws on the results of qualitative interview studies with decision makers (*n* = 22) from various HSCOs and patients (*n* = 25) [9,10]. The questionnaire included instruments referring PCC implementation in the daily practice of the HSCOs, its relevance, and also the HSCO’s structures, processes, strategies, culture and climate, and external influences [26]. This article refers to the measurement of PCC implementation and organizational communication climate. The measure used to operationalize PCC implementation was developed within the study “Assessment of patient-centeredness through patient-reported experience measures (ASPIRED)” [27] and was derived from the integrative model of patient-centeredness [6]. This generic model is based on literature extracting 15 dimensions of PCC within three categories. One category refers to general principles including four dimensions (patient as a unique person, biopsychosocial perspective, clinician–patient relationship, essential characteristics of the provider). A second category refers to enablers including five dimensions (integration of medical and non-medical care, teamwork and teambuilding, access to care, coordination and continuity of care, provider–patient communication). A third category refers to activities including six dimensions (patient information, patient involvement in care, involvement of family and friends, patient empowerment, physical support, emotional support) [28,29]. The model was validated by assessing the perspectives of various healthcare stakeholders on its relevance and clarity [29]. The measure is based on an expert validation study in which the relevance and implementation of PCC were assessed from the perspective of patients relating to the 15 dimensions. In our study, one dimension from the original instrument was dropped, as it concerned the consideration of provider´s characteristics, and in this study, providers themselves were asked. A further dimension (“considerations of spiritual needs”) was included based on the previous interview studies [9,10]. Participants were asked “To what extent does your organization succeed in successfully implementing or considering the following aspects in everyday care?”. The nine response options ranged from “not at all” (1) over “partly” (5) to “to a great extent” (9) and were aggregated to a composite mean score for the analysis. Cronbach’s alpha of the scale in the population studied was 0.83 [26].

The independent variable communication climate consists of four items measuring the extent of open and constructive communication, cooperation, and inclusion (see Appendix A, Table A1) [30]. The four response options ranged from “strongly disagree” (1) to “strongly agree” (4). The scale has been used in other studies in German organizations [31]. Cronbach’s alpha of the scale in the population studied was 0.84 [26]. HSCO type and the number of employees were considered as covariates due to their correlation with communication climate and PCC implementation.

### 2.4. Data Analysis

In a first step, descriptive analyses of the three categories of PCC implementation and an aggregated total score of the three categories for the overall sample and for each of the six types of HSCOs were conducted. In a second step, a Kruskal–Wallis test to determine if mean levels of PCC implementation were different between the six HSCO types were performed. Post hoc pair-wise test was applied and is based on Dunn’s test, which is described as the appropriate procedure following a Kruskal–Wallis test [32]. In a third step, linear regression modeling to examine associations between PCC implementation and communication climate was used. Two models were estimated. The first model (M1) examined the bivariate association between communication climate and PCC implementation. The second model (M2) added the type of HSCO and the number of employees to test whether the association between communication climate and PCC implementation is affected by these factors. Using interaction analyses, Model 2 was extended by an interaction term between communication climate and HSCO type to test whether the association between communication climate and PCC implementation varies by HSCO. All reported confidence intervals and *p*-values of the regression analyses are based on robust standard errors adjusted for heteroskedasticity [33]. The explanatory strength of communication climate for variations in PCC implementation was assessed with McFadden’s pseudo R^2^. McFadden’s R^2^ ranges from 0 to 1, with higher values indicating a higher explanatory strength. Analyses were performed with Stata 16.0 (StataCorp, College Station, TX, USA).

## 3. Results

### 3.1. Participants

Of 1790 organizations, 237 provided responses. Cases with missing information in variables used for this study were excluded from the analyses. Complete information was available for *n* = 215 observations.

The response rate and total number of HSCOs varies between organizational types (Table 1). The sample of *n* = 215 includes 70% women, 78% are between 46 and 65 years old (range 26 to <65), and 96% work in direct care contact with patients (for sample demographics see Table A2 of the Appendix B). Some of those contacted fed back reasons for non-response. These included a lack of time for participation as well as structural features, such as HSCO with only one or two persons, which made it difficult to answer questions referring to aspects of internal communication. Especially in the group of psychotherapists, the results show that there is a high number of individual practices without employed staff. In the group of general practitioners (GP) and cardiological/internal specialists, some practices also have a very small size. In these two groups, there was a higher number of missing values concerning questions on aspects of communication.

### 3.2. Implementation of PCC in the Total Sample and by Types of HSCOs

In total, the HSCOs show high mean values for all 15 dimensions of self-reported PCC implementation (Table 2). However, there were significant differences in the implementation status between the types of HSCO in terms of overall PCC, principles, and activities (Table 2).

For the overall implementation of PCC, lowest values for PCC implementation were reported for hospitals and highest for psychotherapy practices. The post hoc test shows significantly lower levels for general practitioners compared to outpatient nursing/hospice services (*p* = 0.021) and psychotherapy practices (*p* < 0.001). Psychotherapy practices have significantly higher levels of overall PCC implementation compared to hospitals (*p* = 0.021).

Among the three categories of PCC, dimensions of principles of PCC were most pronounced. The highest value for principles was reported for psychotherapeutic practices, the lowest value for hospitals. The post hoc test revealed significantly lower values for hospitals compared to outpatient nursing/hospice services (*p* = 0.034), outpatient nursing/hospice services (*p* = 0.026), and to psychotherapy practices (*p* < 0.001). Psychotherapy practices also showed significantly higher values than inpatient nursing facilities/hospices (*p* < 0.001), rehabilitation facilities (*p* < 0.001), GP and cardiological/internal specialists (*p* < 0.001), and outpatient nursing/hospice services (*p* < 0.001). Among the dimensions of principles, the implementation of a trustful relationship between patients and providers was perceived to succeed best.

In terms of activities of PCC, the results indicate highest values in psychotherapy practices and outpatient nursing/hospice services, and lowest values for rehabilitation facilities. The post hoc test shows significantly higher values for psychotherapy practices compared to hospitals (*p* = 0.034), rehabilitation facilities (*p* = 0.038), and GP and cardiological/internal and specialists (*p* < 0.001). Moreover, general practitioners and specialists have significantly higher values compared to rehabilitation facilities (*p* = 0.006). Among the dimensions of activities, respondents perceived the involvement of family and friends to be least successful and the support of mental well-being the most.

In terms of the enablers that are essential for providing PCC, inpatient nursing facilities/hospices showed the highest values and GP and cardiological/internal specialists and psychotherapy practices lowest. The post-hoc test shows significant higher levels for inpatient nursing facilities/hospices compared to GP and cardiological/internal specialists (*p* = 0.005) and psychotherapy practices (*p* = 0.008). Among the enablers, respondents perceive the consideration of spiritual needs to be least implemented and the appropriate communication with patients to be most successful.

### 3.3. Communication Climate as Precondition for PCC Implementation

The mean value for the open communication scale was 3.3 (range: 1 to 4) across organizational types, with significant differences between HSCO types (lowest in rehabilitation facilities (2.9), highest in psychotherapy practices (3.6) (results shown in Appendix A, Table A1). Table 3 shows the results of the linear regression of PCC implementation by communication climate.

Model 1 (M1) indicates bivariate associations of communication climate with the three categories of PCC (see Table 3). For all categories, a significant association between communication climate and PCC implementation was observed. Model 2 (M2) was based on M1 and also included HSCO type and number of employees as control variables. Results indicate that associations of communication climate with the categories of PCC implementation outcomes were still significant and only slightly affected by the control variables. The interaction analyses in Model 3 (Appendix C, Table A3) shows that associations between communication climate and categories of PCC implementation were significantly lower in psychotherapy practices compared to inpatient nursing facilities/hospices. The adjusted R^2^ in M1 showed high explanatory strength of communication climate for variations in the overall PCC implementation, principles and activities. The highest explanatory strength was observed in M2 for variations in the category principles of PCC.

## 4. Discussion

Our study examined the status of PCC implementation within diverse HSCOs in Cologne, Germany and analyzed the communication climate as a precondition of successful implementation. From the perspectives of 215 decision makers from six different types of health and social care organizations, our study revealed that the participating HSCOs perceive to already succeed in the overall implementation of PCC. Previous findings on the implementation of patient-centered care and its preconditions insufficiently account for the organizational context to explain differences.

This study revealed that the reported status of implementation significantly differed by types of HSCO and in terms of the three categories of PCC. Dimensions of principles of PCC were most pronounced. This is in line with research findings that emphasize a patient-centered principles and attitudes of providers as the basis for activities to promote PCC and the transformation towards a “holistic” approach that takes into account the biopsychosocial needs of patients [34].

According to the participants, the consideration of spiritual needs and the involvement of family and friends were the least implemented aspects of PCC. This may be due to the fact that providers, in view of limited resources, especially time, focus on their core activities and those aspects of care that they believe are most important to the patient. In most cases, these are perceived to be physical and symptom-related aspects [35]. The involvement of families and friends could then hinder their work. This underlines the dominance of a medical model of care. It might also mean that providers are not prepared to take a more comprehensive, psychosocial perspective and are not in a position to manage more holistic aspects of care. Given the fact that an increasing number of chronically ill patients also have psychological problems and individual needs, this represents a deficit in care [36].

In general, hospitals reported the lowest values of PCC implementation and psychotherapy practices the highest. Differences in provider´s patient-centered principles by HSCO type, as precondition for other PCC dimensions, have been known over time [37,38,39], but are insufficiently explained. As hospitals differ the most from psychotherapeutic practices in terms of their types and diversity of professional trainings, mission of care, structures, and their resources, the question about organizational preconditions which help to implement PCC arises. Hospitals have certain preconditions that impede the implementation of PCC and may explain the lower values of the implementation status in our study. According to O’Lealry et al. [40], these preconditions include for example not having a prior exchange of information with patients´ providers, the high complexity and pace of clinical care, and deficits in interprofessional cooperation.

In our study, higher values of PCC implementation occurred in psychotherapy practices especially referring to principles. This may due to the fact that psychotherapy practice is generally based on a holistic approach of care with emphasis on the therapeutic relationship [41]. PCC is a familiar term and concept in psychotherapy for quite a while and builds on work from Roger [42]. Nevertheless, the literature points out that there is a need for improvement of PCC in psychotherapy, especially regarding the reduction of complexity in care structures of psychotherapy, which contradicts patient-centered care [41,43]. This is confirmed in our study, since psychotherapy practices, in comparison to other types of organizations, indicated the lowest level of cooperation with other providers, access to care, and inclusion of additional services. However, this cannot be solved by individual providers and requires cross-sectoral approaches of care. This indicates a need for improvement, as another study also identified continuity of care across sectors and providers as one of the most important characteristics of PCC [9]. Another point to consider is that psychotherapists in our study are most likely to rate their own actions only, whereas decision makers in hospitals, for example, rate over several hundred employees and therefore have a greater gap between their own actions and the overall assessment.

The association between PCC implementation and the communication climate has been rarely researched for various HSCO contexts. This study identified communication climate as a precondition of PCC implementation with high explanatory strength, especially in terms of the basis category principles. The association between communication climate and PCC implementation outcomes was only slightly affected by number of employees and was lower in psychotherapeutic practices. This result could be based on the fact that the importance of communication climate for PCC increases with the size of the HSCO. Psychotherapy practices have lowest numbers of employees and rarely cooperate interprofessionally. Hospitals have the highest number of employees, characterized by a high variation of professional groups and interests. These characteristics were found to facilitate coordination and communication problems [16] and may further explain the lower values for PCC in hospitals. In hospitals, communication takes place between various professional groups and between several and alternating individuals. This might be a disadvantage to build a positive, open communication climate based on social capital, that enables social cooperation, psychological safety, and trustful debates over problems at work [12,13], characterized by an understanding of roles and responsibilities, agreement on the approach to care, absence of power dynamics, proper communication patterns, and constant exchange of information [14]. Similarly, hospitals have more pronounced hierarchies due to the diversity of professional groups and areas of responsibility. This also hinders a positive communication climate [14]. Consequently, leadership culture becomes an essential starting point for increasing positive communication climate and therefore PCC implementation [4,44].

In contrast, the communication between psychotherapists is rather for professional exchange, e.g., for supervision [9], than for coordinating patient care, since patients are usually only cared for by one therapist and coordination is not required. This rather beneficial form of communication can lead to healthy relationships and well-being, which is associated with higher PCC implementation in different HCSO settings [9,19].

### Study Limitations

Our results need to be seen in light of several limitations of this study. Our sample might suffer from selection bias for several reasons. (1) Participants might have had a higher intrinsic motivation and interest in the research topic than non-participants and might also be more likely to engage in activities that foster PCC. (2) Despite the application of several strategies shown to increase survey response rates, the response rate of 13% remained low and might have contributed to a selection bias. Especially in HSCOs with smaller numbers of employees, response rates were low. However, response rates in organizational surveys are known to be rather low and decreased within the last decades [45]. A further explanation for the lower response rate might be the method of data collection via postal surveys, although this method is considered the most effective in the target group of health professionals [46]. (3) The number of organizations surveyed differs between the types of organizations with lowest numbers for rehabilitation facilities and hospitals, as there are fewer of them in the city (Cologne) than other types. The comparability between the types of HSCO is partially limited, e.g., in psychotherapy practices. There are also varying numbers of employees within the organizations limiting the comparability of answers given. However, this has been taken into account in the best possible way by controlling for the number of employees in the analyses. The outcomes and effects for each type of organization were analyzed separately and controlled for effects of the organization type relating to the correlations. Nevertheless, it is so far rare to compare these different types of HSCOs at all, and results can provide new findings. (4) Our target group were decision makers in leading positions so that differences in perspectives across hierarchies cannot be identified through this study. (5) Finally, all scales were assessed using self-reports, and common method variance may have biased the results [47].

As this study focused on the German healthcare system and is geographically restricted to the city of Cologne, the results may not be directly transferable to other healthcare systems. However, efforts have been made to survey the implementation of PCC in Cologne. The results are potentially transferable to structurally and demographically comparable metropolitan regions in Germany. Nevertheless, future research should investigate whether the findings are similar in other regions, especially rural areas.

## 5. Conclusions

The implementation status of patient-centered care was perceived as high but differed between the various types of organizations. Communication climate was identified as a precondition of PCC implementation with high explanatory strength. Improving the communicative skills of health and social care providers and building a culture of social action and open, positive communication within the HSCO have shown to be crucial starting points for initiating the redesign of health and social care towards more patient-centeredness. Especially in HSCOs with a higher number of employees, strategies to create an open communication climate are needed to promote PCC. Future studies are needed to validate the explorations of this work and to employ in-depth analyses to unravel systematic differences between types of HSCOs in terms of PCC implementation. In order to increase a positive, open communication climate, social capital must be strengthened by promoting a leadership culture that reduces hierarchies and power dynamics and allows a cooperative and appreciative cooperation to develop [4,44]. Information and communication technologies can help to improve communication processes, especially between interdependent hierarchical levels [48]. Specific training programs or communication tools [49] can also improve communication behavior, for example by promoting an open feedback culture [50]. The implementation and success of such strategies should be investigated and evaluated in future studies.

## Figures and Tables

**Table 1:** Response rates of the survey by type of organization and proportion of the total response.

Organizational Type	Contacted	Respondents	% of Return within Organizational Type	Analysis Sample *
Inpatient nursing facilities/hospices	86	19	22.1%	19
Hospitals	42 **	15 ***	35.7%	11
Rehabilitation facilities	13	6	46.2%	6
GP and cardiological/internal specialists	665	79	11.9%	73
Outpatient nursing/palliative services	177	22	12.4%	22
Psychotherapy practices	807	96	11.9%	84
Total	1790	237	13.2%	215

* Sample after drop-out due to missing values in variables used; ** Individuals from 22 hospitals; *** Individuals from 11 hospitals.

**Table 2:** Mean values of PCC implementation for the total sample and for HSCOs separately (range: 1−9, *n* = 215).

Outcomes of PCC Implementation	Mean Value for Total Sample	Inpatient Nursing Facilities/Hospices	Hospitals	Rehabilitation Facilities	GP and Cardiological/Internal Specialists	Outpatient Nursing/Hospice Services	Psychotherapy Practices	Kruskal–Wallis Test $X^{2}\;(\mathrm{df}):\;p-\mathrm{Value}$
**Overall PCC implementation**	7.36	7.48	7.10	7.19	7.17	7.58	7.64	15.434 (5): 0.009
**Principles**	7.64	7.39	6.85	7.50	7.58	7.79	8.71	79.208 (5): < 0.001
Uniqueness of each patient	7.47	7.26	6.45	7.50	7.33	7.55	8.73	
Consideration of personal circumstances	7.52	6.95	6.82	7.50	7.45	7.82	8.56	
Trustful relationship between patient and provider	7.92	7.95	7.27	7.50	7.96	8.00	8.82	
**Activities**	7.32	7.30	7.20	6.97	7.12	7.65	7.66	18.816 (5): 0.002
Collaboration as equal partners and involvement in decision-making	7.13	6.53	6.09	7.33	6.94	7.68	8.21	
Involvement of family and friends	6.44	6.74	7.27	5.00	6.49	7.55	5.56	
Support of physical well-being	7.52	8.11	7.64	7.17	7.23	8.18	6.76	
Support of mental well-being	7.81	7.63	7.55	7.50	7.71	7.64	8.80	
Personally tailored information	7.35	7.00	6.91	7.17	7.21	7.32	8.48	
Empowerment of patients	7.67	7.89	7.73	7.67	7.04	7.55	8.13	
**Enablers**	7.27	7.71	7.14	7.25	7.02	7.42	7.09	8.603 (5): 0.126
Consideration of spiritual needs	6.04	7.53	6.36	4.83	5.52	5.86	6.12	
Access to care	7.55	8.11	7.18	8.00	7.43	7.77	6.78	
Integration of additional healthcare elements	7.18	7.89	7.17	7.33	6.63	7.59	6.49	
Good planning of care	7.71	7.58	7.45	8.17	7.40	7.95	7.72	
Teamwork of providers	7.30	7.42	7.55	7.83	7.19	7.18	6.65	
Appropriate communication with patients	7.84	7.74	7.09	7.33	7.99	8.14	8.77	

**Table 3:** Linear regression models of PCC implementation by communication climate (*n* = 215).

Outcomes of PCC Implementation	M1 (Bivariate)		M2 (+ Control Variables)	
	ß (CI−95%)	Adj. R2	ß (CI−95%)	Adj. R2
**Overall PCC implementation**	0.546 ***	0.1436	0.490 ***	0.2114
	(0.312; 0.780)		(0.241; 0.739)	
**Principles**	0.724 ***	0.1461	0.410 **	0.4092
	(0.419; 1.029)		(0.158; 0.662)	
**Activities**	0.622 ***	0.1562	0.552 ***	0.2254
	(0.423; 0.821)		(0.349; 0.755)	
**Enabler**	0.380 *	0.0441	0.467 *	0.1188
	(0.074; 0.687)		(0.106; 0.829)	

Notes: * *p* ≤0.05; ** *p* < 0.01; *** *p* < 0.001. Control variables were HSCO type and number of employees. For full details of the results see Appendix C Table A3.

## Appendix A

**Table A1:** Mean values of communication climate for the total sample and for HSCOs separately (range: 1−4, *n* = 215).

Outcomes of PCC Implementation	Mean Value for Total Sample	Inpatient Nursing Facilities/Hospices	Hospitals	Rehabilitation Facilities	GP and Cardiological/Internal Specialists	Outpatient Nursing/Hospice Services	Psycho-therapy Practices	Kruskal–Wallis Test $X^{2}\;(\mathrm{df}):\;p-\mathrm{Value}$
Communication Climate	3.35	3.16	2.91	2.88	3.29	3.25	3.55	26.504 (5): <0.001
Problems are addressed openly	3.31	3.21	2.93	2.83	3.22	3.27	3.49	12.868 (5): 0.025
Welcome of constructive criticism	3.46	3.32	2.86	2.83	3.47	3.36	3.61	15.058 (5): 0.010
Good separation of factual and personal issues in meetings	3.30	3.05	3.00	3.00	3.15	3.18	3.55	16.032 (5): 0.007
Participation of employees in important decision-making	3.32	3.05	2.86	2.83	3.32	3.18	3.51	15.831 (5): 0.007

## Appendix B

**Table A2:** Sample demographics (*n* = 215).

Age		*n*	(%)
	26−35 years	6	(2.8)
	36−45 years	42	(19.5)
	46−55 years	75	(34.9)
	56−55 years	75	(34.9)
	> 65 years	17	(7.9)
Gender			
	Males	64	(29.8)
	Females	151	(70.2)
Organization type			
	Inpatient nursing facilities/hospices	19	(8.8)
	Hospitals	11	(5.1)
	Rehabilitation facilities	6	(2.8)
	GP and cardiological/internal specialists	73	(33.9)
	Outpatient nursing/hospice services	22	(10.2)
	Psychotherapy practices	84	(39.1)
Ever been active in direct patient care			
	No	7	(3.3)
	Yes	207	(96.3)
	Missing data	1	(0.5)
If not active in direct patient care: in the past, active in direct patient care			
	No	7	(23.3)
	Yes	22	(73.3)
	Missing data	1	(3.3)
Field of activity			
	Nursing	18	(8.4)
	Medical	72	(33.5)
	Therapeutic	92	(42.8)
	Management and Administration	24	(11.2)
	Another	4	(1.9)
	Missing data	5	(2.3)
Professional background			
	Medicine	74	(31.2)
	Psychology	104	(43.9)
	Nursing	34	(14.4)
	Social work	7	(3.0)
	Management & Finances	8	(3.4)
	Another	6	(2.5)
	Missing data	4	(1.7)
Leadership position			
	No	98	(45.6)
	Yes	117	(54.4)
Self-employed			
	No	61	(28.4)
	Yes	154	(71.6)

## Appendix C

**Table A3:** Linear regression for PCC implementation by communication climate.

Outcomes of PCC Implementation	Total PCC			Principles			Activities			Enabler		
	M1	M2	M3	M1	M2	M3	M1	M2	M3	M1	M2	M3
	β ‘95%-CI	β ‘95%-CI	β ‘95%-CI	β ‘95%-CI	β ‘95%-CI	β ‘95%-CI	β ‘95%-CI	β ‘95%-CI	β ‘95%-CI	β ‘95%-CI	β ‘95%-CI	β ‘95%-CI
Communication climate	0.546 ***	0.490 ***	1.082 ***	0.724 ***	0.410 **	1.137 ***	0.622 ***	0.552 ***	1.118 ***	0.380 *	0.467 ***	0.950 ***
	0.312; 0.780	0.241; 0.739	0.559; 1.606	0.419; 1.029	0.158; 0.662	0.409; 1.865	0.423; 0.821	0.349; 0.755	0.623; 1.752	0.074; 0.687	0.106; 0.829	0.384; 1.517
HSCO [reference - Inpatient nursing facilities/hospices]:												
Hospitals		−0.173	−0.113		−0.058	2.034		−0.450	0.656		−0.047	−1.390
		−0.417; 0.762	−2.498; 2.724		−0.893; 1.009	−1.801; 5.870		−1.053; 1.153	−1.901; 3.213		−0.701; 0.606	−4.554; 1.775
Rehabilitation facilities		−0.221	2.516		0.155	3.795		−0.237	1.948		−0.394	2.443
		- 0.851; 0.409	−0.717; 5.748		−0.511; 0.820	−0.958; 6.633		−1.014; 0.540	−1.893; 5.789		−1.039; 0.252	−1.007; 5.893
GP and cardiological/internal specialists		−0.468 *	1.087		−0.034	2.126		−0.347	1.351		−0.842 ***	0.305
		−0.866; −0.069	−1.196; 3.370		−0.527; 0.595	−0.828; 5.080		−0.788; 0.094	−0.873; 3.576		−1.249; −0.435	−2.574; 3.184
Outpatient nursing/hospice services		−0.009	1.598		0.289	2.497		0.235	2.348		−0.402	0.390
		−0.470; 0.451	−1.117; 4.313		−0.316; 0.893	−0.726; 5.720		−0.288; 0.758	−0.620; 5.315		−0.900; 0.095	−2.751; 3.549
Psychotherapy practices		−0.129	2.749 **		1.052 ***	4.063 **		0.047	3.051 **		−0.895 ***	1.792
		−0.515; 0.258	0.772; 4.725		0.528; 1.576	1.494; 6.633		−0.377; 0.470	1.038; 5.064		−1.321; −0.469	−0.805; 4.390
Number of employees		−0.001 **	−0.001 *		−0.001	−0.001		−0.001 *	−0.001		−0.001 **	−0.001
		−0.002; −0.000	−0.002; −0.000		−0.003; 0.000	−0.003; 0.000		−0.002; −0.000	−0.002; 0.000		−0.002; −0.000	−0.002; 0.000
Interaction term between communication climate and...												
... Hospitals			0.013			−0.625			−0.069			0.414
			−0.770; 0.796			−1.821; 0.571			−0.844; 0.706			−0.482: 1.311
... Rehabilitation facilities			−0.890			−1.195 **			−0.694			−0.933
			−1.912; 0.133			−2.090; −0.299			−1.924;0.536			−1.992; 0.126
... GP and cardiological/internal specialists			−0.491			−0.665			−0.537			−0.360
			−1.187; 0.205			−1.552; 0.223			−1.215; 0.140			−1.225; 0.504
...Outpatient nursing/hospice services			−0.508			−0.700			−0.665			−0.254
			−1.368; 0.353			−1.692; 0.293			−1.573; 0.243			−1.256; 0.747
... Psychotherapy practices			−0.871 **			−0.929 *			−0.913 **			−0.803 *
			−1.469; −0.247			−1.696; −0.161			−1.520; −0.305			−1.568; −0.038
Constant	5.592 ***	6.038 ***	4.149 ***	5.558 ***	6.208 ***	3.911 ***	5.316 ***	5.660 ***	3.636 ***	5.888 ***	6.336 ***	4.782 ***
	4.792; 6.391	5.162; 6.915	2.451; 5.847	4.491; 6.624	5.236; 7.180	1.500; 6.323	4.637; 5.994	4.885; 6.436	1.777; 5.495	4.843; 6.933	5.130; 7.542	2.950; 6.613
Adjusted R^2^	0.1396	0.2114	0.2457	0.1461	0.4092	0.4267	0.1562	0.2254	0.2539	0.0441	0.1188	0.1469
*n*	215	215	215	215	215	215	215	215	215	215	215	215

Notes: * *p* ≤ 0.05; ** *p* < 0.01; *** *p* < 0.001.
